# External Validation of a Nomogram to Predict Survival and Benefit of Concurrent Chemoradiation for Stage II Nasopharyngeal Carcinoma

**DOI:** 10.3390/cancers13174286

**Published:** 2021-08-25

**Authors:** Pui-Lam Yip, Shing-Fung Lee, Cheuk-Wai Horace Choi, Po-Chung Sunny Chan, Ka-Wai Alice Cheung, Chung-Hang James Chow, Ka-Man Cheung, Wing-Yu Jessica Lai, Ho-Fun Victor Lee, Ka-On Lam, Chi-Leung Chiang, Chun-Yin Edwin Wong, Ming-Chun Darren Poon, Macy Tong, Kwok-Hung Au, Wai-Tong Ng, Kai-Cheong Roger Ngan, Wing-Mui Anne Lee, Yuk Stewart Tung

**Affiliations:** 1 Department of Clinical Oncology, Tuen Mun Hospital, Hospital Authority, Hong Kong, China; ypl456@ha.org.hk (P.-L.Y.); pcchan@ha.org.hk (P.-C.S.C.); ckw045@ha.org.hk (K.-W.A.C.); chiangcl@hku.hk (C.-L.C.); tungys@ha.org.hk (Y.S.T.); 2 Department of Clinical Oncology, The University of Hong Kong, Hong Kong, China; hcchoi@hku.hk (C.-W.H.C.); vhflee@hku.hk (H.-F.V.L.); lamkaon@hku.hk (K.-O.L.); ngwt1@hku.hk (W.-T.N.); rkcngan@hku.hk (K.-C.R.N.); awmlee@hku.hk (W.-M.A.L.); 3 Department of Clinical Oncology, University of Hong Kong-Shenzhen Hospital, Hong Kong, China; 4 School of Public Health, Li Ka Shing School of Public Health, The University of Hong Kong, Hong Kong, China; 5 Department of Clinical Oncology, Queen Elizabeth Hospital, Hospital Authority, Hong Kong, China; cch932@ha.org.hk (C.-H.J.C.); ckm792@ha.org.hk (K.-M.C.); akhz01@ha.org.hk (K.-H.A.); 6 Department of Oncology, Princess Margaret Hospital, Hospital Authority, Hong Kong, China; lwy627a@ha.org.hk; 7 Department of Clinical Oncology, Pamela Youde Nethersole Eastern Hospital, Hospital Authority, Hong Kong, China; wcy511@ha.org.hk; 8 Department of Clinical Oncology, Prince of Wales Hospital, Hong Kong, China; mc_poon@clo.cuhk.edu.hk (M.-C.D.P.); tongm@ha.org.hk (M.T.)

**Keywords:** nasopharyngeal carcinoma, chemotherapy, radiotherapy, nomogram, survival

## Abstract

**Simple Summary:**

The optimal treatment strategy (concurrent chemoradiation (CCRT) vs. radiotherapy alone) for stage II nasopharyngeal carcinoma (NPC) in the intensity-modulated radiotherapy (IMRT) era is controversial across guidelines. A nomogram by Sun et al. was published to predict the overall survival (OS) benefit of CCRT based on a patient’s clinical parameters. Using the cohort from the Hong Kong NPC1301 study, we evaluated the external validity of the nomogram and the associations between the proposed clinical factors and OS among stage II NPC patients. Use of CCRT was an insignificant predictor for OS. The nomogram lacked the predictive accuracy and should be interpreted with caution.

**Abstract:**

A nomogram was recently published by Sun et al. to predict overall survival (OS) and the additional benefit of concurrent chemoradiation (CCRT) vs. radiotherapy (RT) alone, in stage II NPC treated with conventional RT. We aimed to assess the predictors of OS and to externally validate the nomogram in the IMRT era. We analyzed stage II NPC patients treated with definitive RT alone or CCRT between 2001 and 2011 under the territory-wide Hong Kong NPC Study Group 1301 study. Clinical parameters were studied using the Cox proportional hazards model to estimate OS. The nomogram by Sun et al. was applied with 1000 times bootstrap resampling to calculate the concordance index, and we compared the nomogram predicted and observed 5-year OS. There were 482 patients included. The 5-year OS was 89.0%. In the multivariable analysis, an age > 45 years was the only significant predictor of OS (HR, 1.98; 95%CI, 1.15–3.44). Other clinical parameters were insignificant, including the use of CCRT (HR, 0.99; 95%CI, 0.62–1.58). The nomogram yielded a concordance index of 0.55 (95% CI, 0.49–0.62) which lacked clinically meaningful discriminative power. The nomogram proposed by Sun et al. should be interpreted with caution when applied to stage II NPC patients in the IMRT era. The benefit of CCRT remained controversial.

## 1. Introduction

Nasopharyngeal carcinoma (NPC) is endemic in Southeast Asia. The age-standardized incidence rates (per 100,000 persons) were 5 in Southeast Asia and 1.6 globally, respectively [1]. According to the Hong Kong Cancer Registry, the crude rate was 11.2 per 100,000 persons in Hong Kong in 2018. Stage II NPC comprises 11.5% and 14.1% of all stages in the seventh and eighth edition AJCC [2,3]. While radiotherapy (RT) is the mainstay of definitive treatment in stage II NPC, the additional benefit of concurrent chemoradiation (CCRT) in the intensity-modulated radiotherapy (IMRT) era remains controversial [4,5,6,7]. The National Comprehensive Cancer Network (NCCN) guideline [8] recommends CCRT with induction or adjuvant chemotherapy for stage II-IVB NPC. On the other hand, the latest CSCO/ASCO guideline [9] recommends the decision on CCRT to be based on the TN subcategory and risk assessment. In contrast, the ESMO/EURACAN guideline [10] suggests that RT alone could be considered if IMRT is used. Treatment outcomes in early NPC have improved remarkably in the IMRT era [5,11,12]. In contrast to the traditional two-dimensional radiotherapy, IMRT utilizes multiple radiation beams and modulated radiation intensities to deliver an adequate dose to the tumor in a conformal shape with high precision, while minimizing radiation spillage to the adjacent critical organs [13].

Recently a nomogram [14] was proposed by Sun et al. to estimate the 5-year and 10-year overall survival (OS) in stage II NPC. It predicted the additional benefit of CCRT based on the data from a landmark randomized controlled trial (RCT) in 2011 [15] that showed CCRT improved OS, progression-free survival, and distant metastasis in stage II NPC treated with a conventional RT technique.

This nomogram is easy to use and consists of clinical parameters commonly reported in cancer staging. It could potentially inform clinicians and patients of disease prognosis and estimate the clinical benefit of CCRT, for shared treatment decisions. In this study, we performed an external validation on the proposed nomogram to review its discrimination and accuracy, to study whether it should be widely adopted in the modern treatment era.

## 2. Materials and Methods

### 2.1. Patients and Treatment

We retrospectively reviewed data from the Hong Kong NPC Study Group (HKNPCSG) 1301 study [16]. The data was based on the Hong Kong Cancer Registry. All clinical data and treatment records were retrieved through the electronic patient record system of the six oncology centers in public hospitals in Hong Kong. All patients underwent physical examination, fiberoptic nasopharyngoscopy, and MRI of the nasopharynx and neck (or CT if contraindicated to MRI) as part of the pretreatment evaluation. They were retrospectively staged according to the seventh edition of the AJCC/UICC staging system [17], and clinical information was validated in the previous study by the principal investigator. Inclusion criteria were similar to the original study [14,15]. Stage II (seventh edition AJCC/UICC) treatment naïve NPC patients, with WHO type II or III histology, aged 70 or under, who underwent definitive IMRT alone, or CCRT, were selected from the database. We excluded patients without staging MRI, and those who received neoadjuvant and/or adjuvant chemotherapy. All patients were treated with IMRT according to their institutional practice. RT details had been reported [16]. Concurrent chemotherapy regimens were commonly cisplatin 30–40 mg/m^2^ weekly, cisplatin 100 mg/m^2^ three-weekly, or for selected patients, carboplatin. The use of concurrent chemotherapy was based on patient factors, the clinicians’ discretion, and the individual center’s protocol. Fiberoptic nasopharyngoscopy was performed at 6–16 weeks after RT completion. Subsequent follow-up schedules followed institutional policies. This study was approved by all individual institution review boards.

The nomogram under study was published in the paper by Sun et al. [14]. We studied the clinical and treatment factors, namely: 1. Age, 2. T category, 3. N category, 4. Treatment group, and calculated the total points for each patient. The predicted probability of OS was estimated from the nomogram. The observed OS was derived from survival analyses in our study cohort.

### 2.2. Statistical Analysis

The primary endpoint was OS, which was defined as the time interval from the start of RT to any cause of death or the date of censoring at the last follow-up. Baseline characteristics were evaluated using a Chi-square test for categorical variables and t-test for continuous data (or Mann–Whitney U test if appropriate). Kaplan–Meier curves were used for survival data. A log-rank test was used to compare the survival between treatment groups and stages. The Cox proportional hazards model was used for univariable and multivariable analyses, and to determine the adjusted hazard ratio with a 95% confidence interval. A *p* < 0.05 was considered statistically significant. Total points were calculated from the nomogram to predict 5-year OS. The discrimination and calibration were evaluated using Harrell’s Concordance Index (C-index) and calibration plot with 1000 times bootstrap resampling. To evaluate the nomogram accuracy, a calibration plot was constructed by grouping patients according to the total points and the corresponding nomogram-predicted 5-year OS probabilities, and then compared with the observed Kaplan–Meier 5-year OS. Perfect prediction accuracy should be on the diagonal line. The C-index and calibration plot were calculated with Stata (Version 16.1, StataCorp, College Station, TX, USA) and R version 3.6.3 (R Foundation for Statistical Computing, Vienna, Austria). Other statistical analyses were performed using SPSS software (Version 21, SPSS Inc., Chicago, IL, USA).

## 3. Results

A total of 589 patients treated in 2001–2011 were identified from the HKNPCSG 1301 study. Of these, 482 patients were included in this study based on the inclusion and exclusion criteria (Supplementary Table S1). Baseline characteristics are shown in Table 1. Of 482 patients, 447 (92.7%) had nonkeratinizing undifferentiated carcinoma. 

Compared with the RT alone group, patients in the CCRT group were more likely to be younger (*p* = 0.02), in category N1 than N0 (*p* < 0.01), and with a more advanced TN category (*p* < 0.01). However, the gender and T category were similar. The median follow-up duration was 86 months. The mean of the total points calculated from the nomogram was 198.53 (standard derivation 58.92); the RT alone group had significantly higher total points than the CCRT group (RT alone: 230.20 vs. CCRT: 143.47; *p* < 0.01).

**Figure 1:**
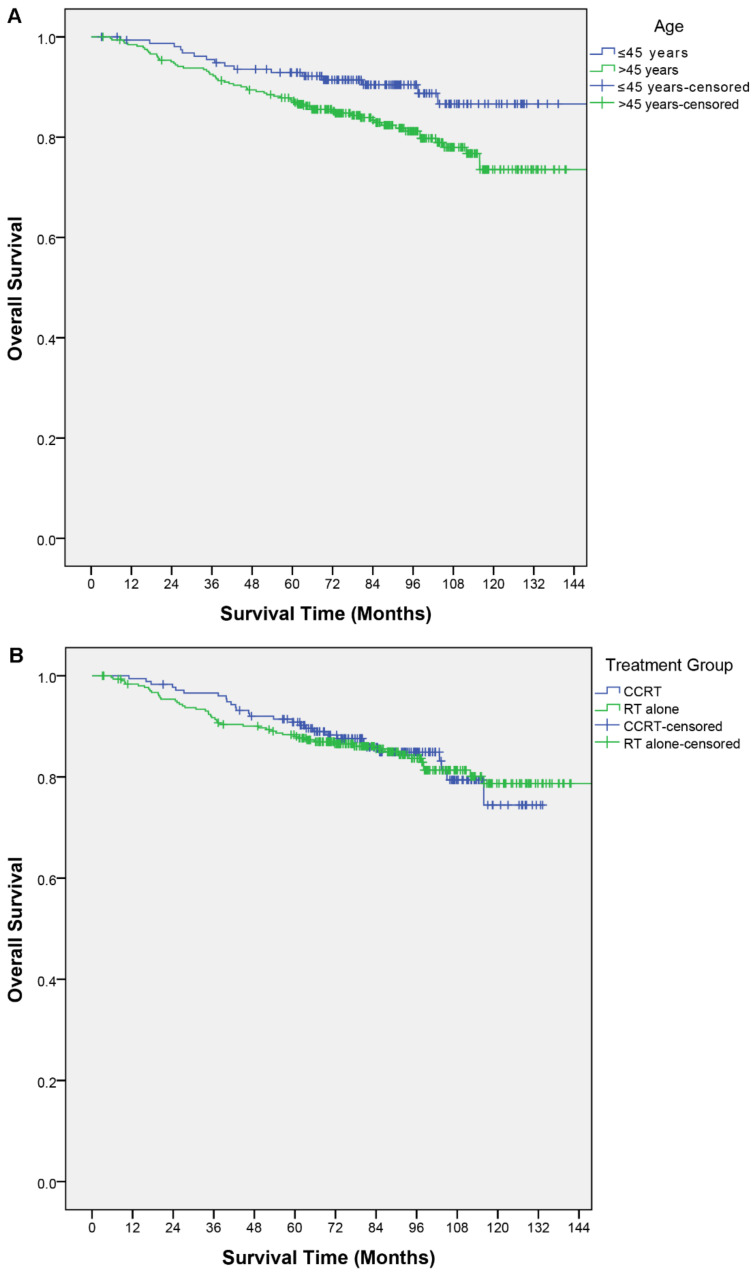

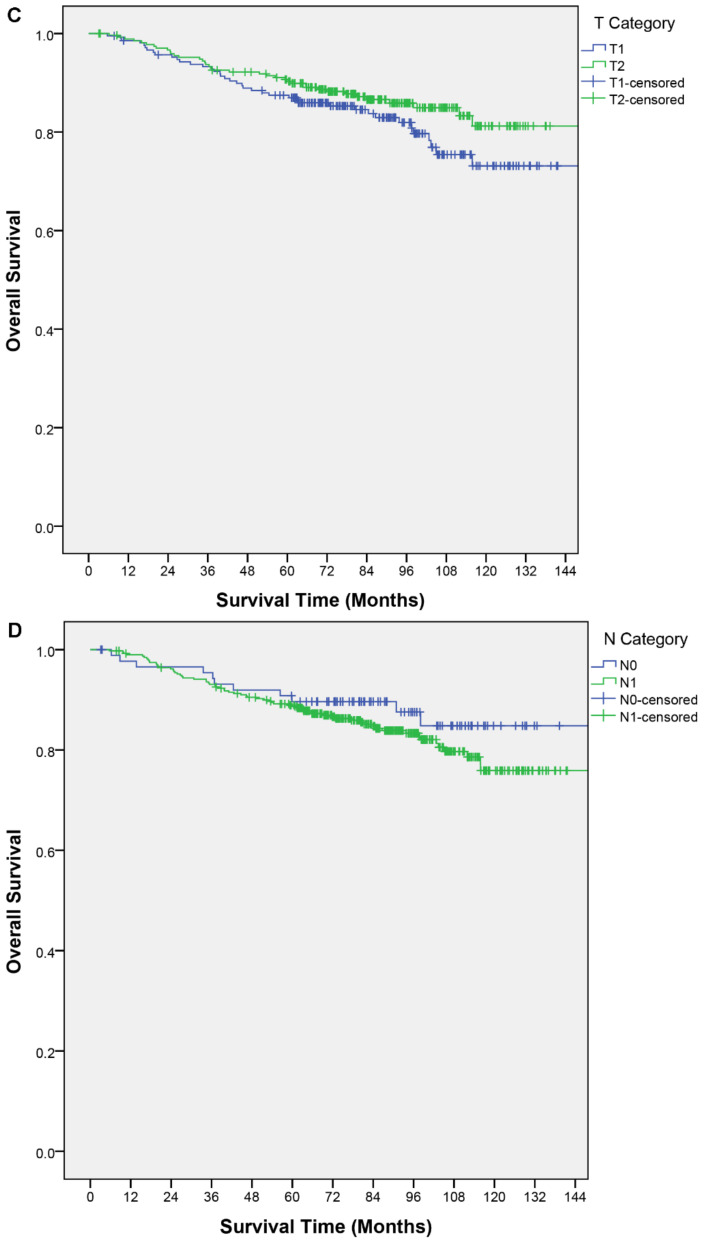

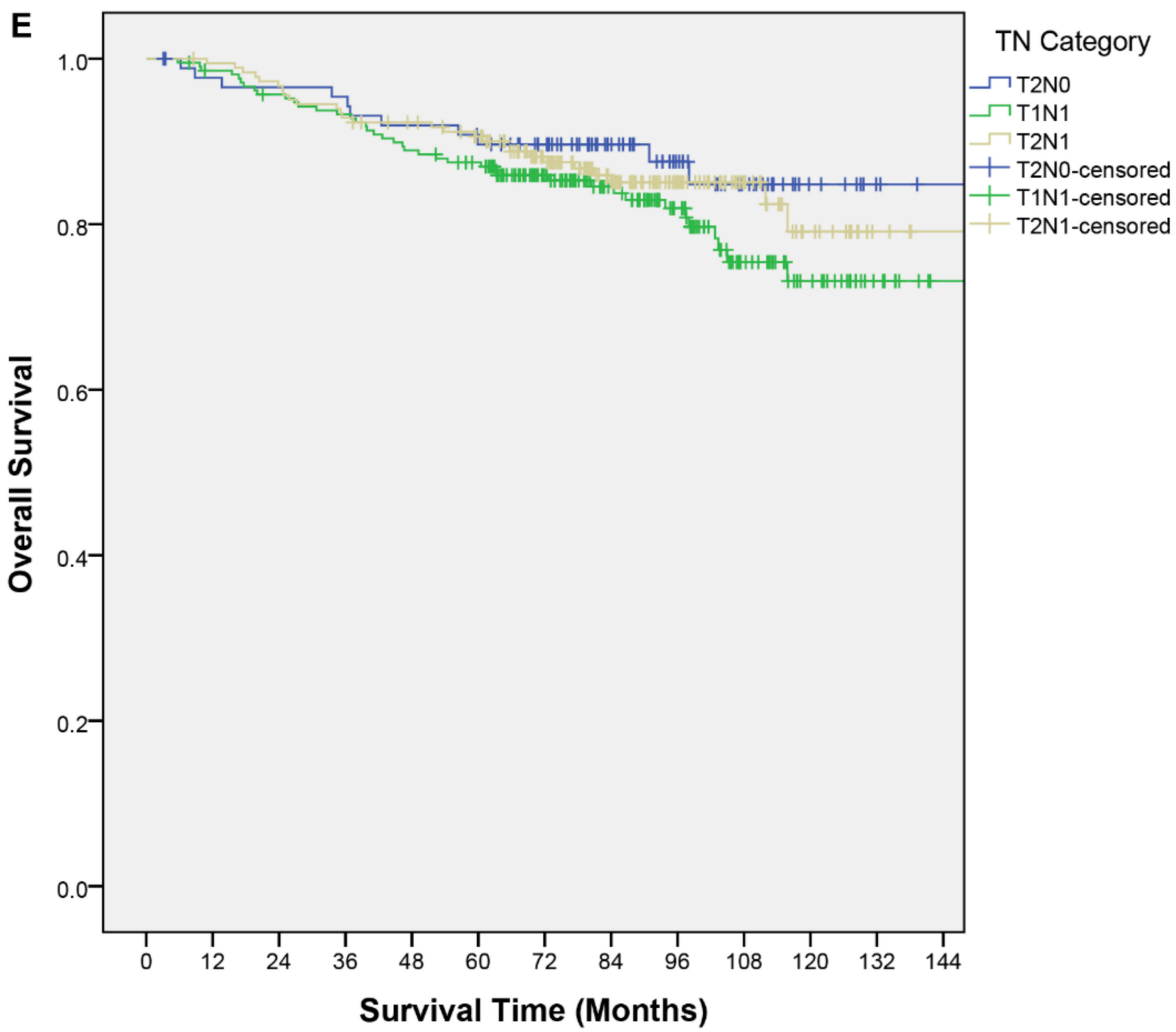
Kaplan–Meier curves of overall survival by (**A**) age, (**B**) treatment group, (**C**) T category, (**D**) N category, (**E**) TN category.

Within the study period, a total of 79 deaths occurred. Twenty-eight in the CCRT group and 51 in the RT alone group. The 5-year and 8-year OS of the overall cohort were 89.0% and 84.1%, respectively. The CCRT group had a 5-year and 8-year OS of 90.8% and 84.9%, while in the RT alone group they were 88.4% and 83.7%, respectively. The log-rank test did not show a significant difference between these two groups (X^2^ = 0.02; *p* = 0.90) (Table 1; Figure 1b).

Clinical factors described in the nomogram and other relevant factors were analyzed for their association with OS. In the univariable analysis, older age was a poor prognostic factor for OS (*p* < 0.01); an age > 45 years had a shorter OS (hazard ratio (HR), 1.97; 95% confidence interval (CI), 1.13–3.40; *p* = 0.02). The T category, N category, and TN category were not significant predictors of OS (Table 2). In the multivariable analysis, an age > 45 years remained the only independent predictor for OS (HR, 1.98; 95% CI, 1.15–3.44; *p* = 0.02). None of the other predictors in the nomogram were significant (Table 3). Figure 1 shows the OS curves by different factor categories.

### Discrimination and Accuracy

**Figure 2:**
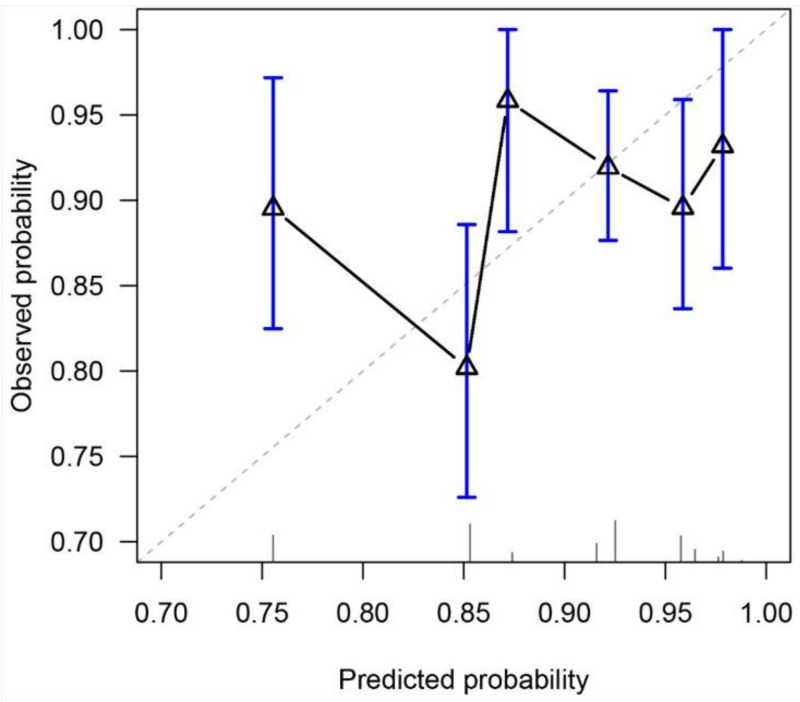
Calibration plot for predicting 5-year overall survival. The diagonal line represents a perfect prediction.

The C-index of the study nomogram was 0.55 (95% CI, 0.49–0.62). A calibration plot (Figure 2) was constructed based on the observed probability of a 5-year OS against the predicted 5-year OS from the nomogram. The scatter plot did not fit with the diagonal line. The calibration slope and intercept were 0.27 (95% CI, 0.23–0.32) and −1.51 (95% CI, −1.64 to −1.38), respectively.

## 4. Discussion

We retrieved data from our Hong Kong territory-wide electronic health database to perform an external validation of a recently published nomogram to predict 5-year OS after curative treatment for stage II NPC patients. However, the result did not support the use of this nomogram to predict OS or make clinical decisions on the use of CCRT in the modern IMRT era.

We reviewed patients with similar inclusion criteria to the original cohort for nomogram construction and validation. Our sample size (N = 482) was equivalent to the original cohorts (N = 199 and 306 for internal and external validation respectively [14]). Only younger patients (i.e., aged ≤ 70 years) were selected, as CCRT, especially a platinum-based regimen, was generally considered less well-tolerated in the older population. Major trials of chemoradiation have also selected only younger populations [18,19,20,21]. Our analysis showed that age was the only significant predictor for OS. T-category, N-category, TN category, or concurrent use of chemotherapy did not predict OS. The concordance index of the nomogram lacked clinically meaningful discriminative power. The calibration plot also did not support prediction accuracy, with concerns on overestimation and overfitted risk estimates.

A phase III RCT showed the survival benefit of CCRT in stage II NPC using 2D conventional RT and staged according to the Chinese 92 staging system [15]. In addition, the nomogram under evaluation in this paper was derived from the patient cohort from that trial after the exclusion of 31 patients that should be regarded as stage III disease in the seventh edition of the AJCC/UICC TNM staging system [14]. Subsequently, there was significant RT technique improvement with IMRT, which further improved local control and survival [22,23,24]. Whether concurrent chemotherapy, in the IMRT era, confers additional survival benefits in stage II NPC had been under much debate. While Luo et al. found an improved survival for CCRT as compared with IMRT in their cohort with predominantly WHO type II histology [25], other phase II trials [26], retrospective cohorts [7,27,28,29,30,31] and meta-analyses [6], [32] have failed to replicate a survival or progression-free survival benefit in the IMRT era. In addition, CCRT had been shown to increase toxicities, including grade three or four neutropenia [6,28], mucositis [15], nausea/vomiting [15] and weight loss [28]. To our knowledge, no subsequent prospective phase III RCT had been published in the IMRT era to confirm the findings from these retrospective analyses.

Stage II NPC had the caveat of being a heterogeneous group of patients with and without lymph node metastasis. Clinicians were generally more inclined to offer concurrent chemotherapy for T2N1 and/or T1N1 patients. Studies had suggested that the T2N1 subgroup had poorer survival outcomes [25,29,33]. The CSCO/ASCO guideline published in late 2020 suggested considering RT alone for T2N0 patients and CCRT for N1, particularly T2N1 patients (eighth AJCC). Our cohort indicated no significant difference in OS between TN categories, and neither the T category nor N category was a useful parameter to make clinical decisions on CCRT. Furthermore, no significant interaction effect was found between the TN category nor the N category and the treatment group in predicting OS. Our result did not concur with other retrospective series [11,29,31,33] that node positivity predicts worse survival. Our cohort is one of the largest. Yet, all the series had a notably different proportion of treatment groups and length of follow-up. Also, like other similar retrospective series, the uneven baseline characteristics in the N and TN categories and the treatment group is an important confounding factor. Prospective studies are needed to confirm our findings. In addition, studies had been conducted to evaluate other parameters, e.g., Epstein–Barr virus (EBV) DNA [34,35,36] and gross tumor volume [37], to refine risk stratification for stage II NPC patients [36]. The result of a phase III noninferiority trial comparing CCRT and RT alone in intermediate-risk NPC with low EBV DNA copies in the IMRT era was eagerly awaited to guide treatment decisions (ClinicalTrials.gov identifier: NCT02135042).

Staging in NPC has evolved over the past decades. The difference in the staging system used in the training cohort (Chinese 1992 staging [38] which was restaged to the seventh AJCC) and validation cohort (seventh AJCC) in Sun et al., as well as in this current cohort (seventh AJCC) should be carefully addressed. The major difference between the two is in the N category. First, patients with a bilateral upper neck LN were restaged to N2 in the seventh AJCC and were excluded from the analyses. Second, patients with lower neck LN or an LN sized 4–6 cm were considered N2 in the Chinese 1992 staging, but regarded as N1 in the seventh AJCC staging. One should exercise extra caution in applying the nomogram in these populations. 

The nomogram is a valuable tool to estimate individualized risk based on patient, disease, and treatment factors. Yet, careful appraisal and application of nomograms are vital. Many nomograms published in the oncology field were solely internally validated or externally validated in patients from the same institute. Issues including over-interpretation, over-fitting, and generalizability across different populations had to be addressed before clinical use [39]. Moreover, rapid advancement in oncological treatment could render previously validated nomograms inaccurate in the modern era. External validation from other institutions is therefore needed to ensure the accuracy and reliability of the decision tool.

A major limitation of this study is that our median follow-up duration was 86 months, shorter than the 120 months in the original trial. We did not analyze the 10-year prediction nomogram due to data limitations. However, the survival curves had already plateaued in our data, and we believed that the influence on survival of the clinical predictors under study would have been apparent within the study period. Despite these limitations, our study has several strengths. We analyzed a reasonably large and homogeneous cohort similar to that in Chen et al. [15] and Sun et al. [14] in terms of patient selection and staging. Moreover, it is a territory-wide study that covered more than 90% of secondary and tertiary care. Incomplete medical information and loss of follow-up were of minor concern. Our cohort and the study outcome are representative of the Hong Kong population in real-world practice.

## 5. Conclusions

The nomogram under study lacked predictive discrimination and accuracy in the modern IMRT era, and it should be used with caution. The benefit of concurrent chemoradiation vs. radiotherapy alone among stage II NPC patients is still controversial. Future research is needed to identify subgroups of stage II NPC patients who may benefit from CCRT. 

## Figures and Tables

**Table 1:** Baseline Characteristics of the Study Cohort, Hong Kong, 2001–2011 (*n* = 482).

Characteristics	Total (*n* = 482)	CCRT (*n* = 176)	RT Alone (*n* = 306)	*p*
Age				
Mean, SD, (range, IQR)		48, 8 (20–68, 43–53)	50, 10 (23–70, 44–57)	0.02
Age ≤ 45, n (%)	158	63 (35.8%)	95 (31.1%)	0.29
Age > 45, n (%)	324	113 (64.2%)	211 (69.0%)	
Sex				
Male, n (%)	337	128 (72.7%)	209 (68.3%)	0.31
Female, n (%)	145	48 (27.3%)	97 (31.7%)	
T category				0.10
T1, n (%)	210	68 (38.6%)	142 (46.4%)	
T2, n (%)	272	108 (61.4%)	164 (53.6%)	
N category				
N0, n (%)	89	17 (9.7%)	72 (23.5%)	<0.01
N1, n (%)	393	159 (90.3%)	234 (76.5%)	
TN category				<0.01
T2N0, n (%)	89	17 (9.7%)	72 (23.5%)	
T1N1, n (%)	210	68 (38.6%)	142 (46.4%)	
T2N1, n (%)	183	91 (51.7%)	92 (30.1%)	
Concurrent chemotherapy				
3-weekly cisplatin, n (%)		62 (35.2%)		
Weekly cisplatin, n (%)		108 (61.4%)		
Carboplatin, n (%)		6 (3.4%)		
Total score by nomogram, mean, SD	198.5, 58.9	143.5, 39.1	230.2, 42.9	<0.01
Follow-up, median, months, IQR	86, 68–106	85, 69–104	86, 68–107	0.66
OS, event		28	51	0.90
5-year OS		90.8%	88.4%	
8-year OS		84.9%	83.7%	

Abbreviations: CCRT, concurrent chemoradiation; IQR, interquartile range; OS, overall survival; RT, radiotherapy; SD, standard deviation.

**Table 2:** Univariable Analysis of Overall Survival, Hong Kong, 2001–2011 (*n* = 482).

Clinical Factors	HR	95% CI	*p*
Age			
≤45 years	reference		
>45 years	1.97	1.13–3.40	0.02
Age (continuous by year)	1.05	1.02–1.07	<0.01
Sex			
Male	reference		
Female	0.77	0.46–1.28	0.31
T Category			
T1	reference		
T2	0.73	0.47–1.14	0.17
N Category			
N0	reference		
N1	1.46	0.77–2.75	0.25
TN Category			
T2N0	reference		0.31
T1N1	1.62	0.83–3.15	0.16
T2N1	1.28	0.64–2.56	0.50
Treatment Group			
RT alone	reference		
CCRT	0.97	0.61–1.54	0.90

Abbreviations: CCRT, concurrent chemoradiation; CI, confidence interval; HR, hazard ratio; RT, radiotherapy.

**Table 3:** Multivariable Analysis of Overall Survival Using Parameters Described in the Study Nomogram, Hong Kong, 2001–2011 (*n* = 482).

Clinical Factors	HR	95% CI	*p*
Age			
≤45 years	reference		
>45 years	1.98	1.15–3.44	0.02
T Category			
T1	reference		
T2	0.77	0.47–1.25	0.29
N Category			
N0	reference		
N1	1.26	0.62–2.55	0.53
Treatment Group			
RT alone	reference		
CCRT	0.99	0.62–1.58	0.96

Abbreviations: CCRT, concurrent chemoradiation; CI, confidence interval; HR, hazard ratio; RT, radiotherapy.

## Data Availability

Due to the nature of this research, participants of this study did not agree for their data to be shared publicly, so supporting data are not available.

## Supplementary Materials

The following are available online at https://www.mdpi.com/article/10.3390/cancers13174286/s1, Table S1: Patient selection from NPC 1301 study.

## References

[1] Nasopharynx. https://gco.iarc.fr/today/data/factsheets/cancers/4-Nasopharynx-fact-sheet.pdf.

[2] Hong Kong Cancer Registry, H.A. Nasopharyngeal Cancer in 2017. https://www3.ha.org.hk/cancereg/pdf/factsheet/2017/npc_2017.pdf.

[3] Hong Kong Cancer Registry, H.A. Nasopharyngeal Cancer in 2018. https://www3.ha.org.hk/cancereg/pdf/factsheet/2018/npc_2018.pdf.

[4] Wu P., Zhao Y., Xiang L., Yang L. (2020). Management of Chemotherapy for Stage II Nasopharyngeal Carcinoma in the Intensity-Modulated Radiotherapy Era: A Review. Cancer Manag. Res..

[5] Lee V.H., Lam K.O., Chang A.T., Lam T.C., Chiang C.L., So T.H., Choi C.W., Lee A.W. (2018). Management of Nasopharyngeal Carcinoma: Is Adjuvant Therapy Needed?. J. Oncol. Pract..

[6] Liu F., Jin T., Liu L., Xiang Z., Yan R., Yang H. (2018). The role of concurrent chemotherapy for stage II nasopharyngeal carcinoma in the intensity-modulated radiotherapy era: A systematic review and meta-analysis. PLoS ONE.

[7] Su Z., Mao Y.P., Tang J., Lan X.W., OuYang P.Y., Xie F.Y. (2016). Long-term outcomes of concurrent chemoradiotherapy versus radiotherapy alone in stage II nasopharyngeal carcinoma treated with IMRT: A retrospective study. Tumour Biol. J. Int. Soc. Oncodevelopmental Biol. Med..

[8] NCCN Clinical Practice Guidelines in Oncology. Head and Neck Cancers. Version 1.2021. https://www.nccn.org/professionals/physician_gls/pdf/head-and-neck.pdf.

[9] Chen Y.-P., Ismaila N., Chua M.L.K., Colevas A.D., Haddad R., Huang S.H., Wee J.T.S., Whitley A.C., Yi J.-L., Yom S.S. (2021). Chemotherapy in Combination With Radiotherapy for Definitive-Intent Treatment of Stage II-IVA Nasopharyngeal Carcinoma: CSCO and ASCO Guideline. J. Clin. Oncol..

[10] Bossi P., Chan A.T., Licitra L., Trama A., Orlandi E., Hui E.P., Halámková J., Mattheis S., Baujat B., Hardillo J. (2020). Nasopharyngeal carcinoma: ESMO-EURACAN Clinical Practice Guidelines for diagnosis, treatment and follow-up(†). Ann. Oncol. Off. J. Eur. Soc. Med Oncol..

[11] Wang L., Miao J., Huang H., Chen B., Xiao X., Zhu M., Liang Y., Xiao W., Huang S., Peng Y. (2021). Long-term Survivals, Toxicities and the Role of Chemotherapy in Early-Stage Nasopharyngeal Carcinoma Patients Treated with Intensity-modulated Radiation Therapy: A Retrospective Study with 15-year Follow-up. Cancer Res. Treat..

[12] Chua D.T., Sham J.S., Kwong D.L., Au G.K. (2003). Treatment outcome after radiotherapy alone for patients with Stage I-II nasopharyngeal carcinoma. Cancer.

[13] Cho B. (2018). Intensity-modulated radiation therapy: A review with a physics perspective. Radiat. Oncol. J..

[14] Sun X.S., Li X.Y., Xiao B.B., Liu S.L., Chen Q.Y., Tang L.Q., Mai H.Q. (2020). Establishment and validation of a nomogram for predicting the benefit of concurrent chemotherapy in stage II nasopharyngeal carcinoma: A study based on a phase III randomized clinical trial with 10-year follow-up. Oral Oncol..

[15] Chen Q.-Y., Wen Y.-F., Guo L., Liu H., Huang P.-Y., Mo H.-Y., Li N.-W., Xiang Y.-Q., Luo D.-H., Qiu F. (2011). Concurrent Chemoradiotherapy vs Radiotherapy Alone in Stage II Nasopharyngeal Carcinoma: Phase III Randomized Trial. JNCI J. Natl. Cancer Inst..

[16] Au K.H., Ngan R.K.C., Ng A.W.Y., Poon D.M.C., Ng W.T., Yuen K.T., Lee V.H.F., Tung S.Y., Chan A.T.C., Sze H.C.K. (2018). Treatment outcomes of nasopharyngeal carcinoma in modern era after intensity modulated radiotherapy (IMRT) in Hong Kong: A report of 3328 patients (HKNPCSG 1301 study). Oral Oncol..

[17] Edge S.B., Compton C.C. (2010). The American Joint Committee on Cancer: The 7th Edition of the AJCC Cancer Staging Manual and the Future of TNM. Ann. Surg. Oncol..

[18] Noronha V., Joshi A., Patil V.M., Agarwal J., Ghosh-Laskar S., Budrukkar A., Murthy V., Gupta T., D’Cruz A.K., Banavali S. (2017). Once-a-Week Versus Once-Every-3-Weeks Cisplatin Chemoradiation for Locally Advanced Head and Neck Cancer: A Phase III Randomized Noninferiority Trial. J. Clin. Oncol..

[19] Kwong D.L., Sham J.S., Au G.K., Chua D.T., Kwong P.W., Cheng A.C., Wu P.M., Law M.W., Kwok C.C., Yau C.C. (2004). Concurrent and adjuvant chemotherapy for nasopharyngeal carcinoma: A factorial study. J. Clin. Oncol. Off. J. Am. Soc. Clin. Oncol..

[20] Lee A.W.M., Ngan R.K.C., Ng W.T., Tung S.Y., Cheng A.A.C., Kwong D.L.W., Lu T.X., Chan A.T.C., Sze H.C.K., Yiu H.H.Y. (2020). NPC-0501 trial on the value of changing chemoradiotherapy sequence, replacing 5-fluorouracil with capecitabine, and altering fractionation for patients with advanced nasopharyngeal carcinoma. Cancer.

[21] Zhang Y., Chen L., Hu G.Q., Zhang N., Zhu X.D., Yang K.Y., Jin F., Shi M., Chen Y.P., Hu W.H. (2019). Gemcitabine and Cisplatin Induction Chemotherapy in Nasopharyngeal Carcinoma. N. Engl. J. Med..

[22] Peng G., Wang T., Yang K.Y., Zhang S., Zhang T., Li Q., Han J., Wu G. (2012). A prospective, randomized study comparing outcomes and toxicities of intensity-modulated radiotherapy vs. conventional two-dimensional radiotherapy for the treatment of nasopharyngeal carcinoma. Radiother. Oncol. J. Eur. Soc. Ther. Radiol. Oncol..

[23] Zhang B., Mo Z., Du W., Wang Y., Liu L., Wei Y. (2015). Intensity-modulated radiation therapy versus 2D-RT or 3D-CRT for the treatment of nasopharyngeal carcinoma: A systematic review and meta-analysis. Oral Oncol..

[24] Co J., Mejia M.B., Dizon J.M. (2016). Evidence on effectiveness of intensity-modulated radiotherapy versus 2-dimensional radiotherapy in the treatment of nasopharyngeal carcinoma: Meta-analysis and a systematic review of the literature. Head Neck.

[25] Luo S., Zhao L., Wang J., Xu M., Li J., Zhou B., Xiao F., Long X., Shi M. (2014). Clinical outcomes for early-stage nasopharyngeal carcinoma with predominantly WHO II histology treated by intensity-modulated radiation therapy with or without chemotherapy in nonendemic region of China. Head Neck.

[26] Huang X., Chen X., Zhao C., Wang J., Wang K., Wang L., Miao J., Cao C., Jin T., Zhang Y. (2020). Adding Concurrent Chemotherapy to Intensity-Modulated Radiotherapy Does Not Improve Treatment Outcomes for Stage II Nasopharyngeal Carcinoma: A Phase 2 Multicenter Clinical Trial. Front. Oncol..

[27] Zhang L.N., Gao Y.H., Lan X.W., Tang J., Su Z., Ma J., Deng W., OuYang P.Y., Xie F.Y. (2015). Propensity score matching analysis of cisplatin-based concurrent chemotherapy in low risk nasopharyngeal carcinoma in the intensity-modulated radiotherapy era. Oncotarget.

[28] Chen K.-H., Zhu X.-D., Li L., Qu S., Liang Z.-Q., Liang X., Pan X.-B., Liang Z.-G., Jiang Y.-M. (2016). Comparison of the efficacy between concurrent chemoradiotherapy with or without adjuvant chemotherapy and intensity-modulated radiotherapy alone for stage II nasopharyngeal carcinoma. Oncotarget.

[29] Guo Q., Lu T., Lin S., Zong J., Chen Z., Cui X., Zhang Y., Pan J. (2016). Long-term survival of nasopharyngeal carcinoma patients with Stage II in intensity-modulated radiation therapy era. Jpn. J. Clin. Oncol..

[30] Xu T., Shen C., Zhu G., Hu C. (2015). Omission of Chemotherapy in Early Stage Nasopharyngeal Carcinoma Treated with IMRT: A Paired Cohort Study. Medicine.

[31] Ding X.C., Fan P.P., Xie P., Fan B.J., Yang J., Jiang L.Y., Bai X.B., Yu J.M., Hu M. (2019). Ten-Year Outcomes Of Intensity-Modulated Radiotherapy (IMRT) Combine With Chemotherapy Versus IMRT Alone For Stage II Nasopharyngeal Carcinoma In The Real-World Study (RWD). Cancer Manag. Res..

[32] Xu C., Zhang L.H., Chen Y.P., Liu X., Zhou G.Q., Lin A.H., Sun Y., Ma J. (2017). Chemoradiotherapy Versus Radiotherapy Alone in Stage II Nasopharyngeal Carcinoma: A Systemic Review and Meta-analysis of 2138 Patients. J. Cancer.

[33] Su S.F., Han F., Zhao C., Chen C.Y., Xiao W.W., Li J.X., Lu T.X. (2012). Long-term outcomes of early-stage nasopharyngeal carcinoma patients treated with intensity-modulated radiotherapy alone. Int. J. Radiat. Oncol. Biol. Phys..

[34] Yao J.J., Zhou G.Q., Wang Y.Q., Wang S.Y., Zhang W.J., Jin Y.N., Zhang F., Li L., Liu L.Z., Cheng Z.B. (2017). Prognostic values of the integrated model incorporating the volume of metastatic regional cervical lymph node and pretreatment serum Epstein-Barr virus DNA copy number in predicting distant metastasis in patients with N1 nasopharyngeal carcinoma. Chin. J. Cancer.

[35] Leung S.F., Chan A.T., Zee B., Ma B., Chan L.Y., Johnson P.J., Lo Y.M. (2003). Pretherapy quantitative measurement of circulating Epstein-Barr virus DNA is predictive of posttherapy distant failure in patients with early-stage nasopharyngeal carcinoma of undifferentiated type. Cancer.

[36] He S.-S., Wang C.-T., Peng Z.-W., Ren Y.-F., Lu L.-X., Chen R.-W., Liang S.-B., Wang Y., Chen Y. (2019). Development and external validation of a nomogram for predicting the overall survival of patients with stage II nasopharyngeal carcinoma after curative treatment. Cancer Manag. Res..

[37] Chen Q.Y., Guo S.Y., Tang L.Q., Lu T.Y., Chen B.L., Zhong Q.Y., Zou M.S., Tang Q.N., Chen W.H., Guo S.S. (2018). Combination of Tumor Volume and Epstein-Barr Virus DNA Improved Prognostic Stratification of Stage II Nasopharyngeal Carcinoma in the Intensity Modulated Radiotherapy Era: A Large-Scale Cohort Study. Cancer Res. Treat..

[38] Min H., Hong M., Ma J., Zhang E., Zheng Q., Zhang J., Zhang J., Zhang F., Su Y., Qiu F. (1994). A new staging system for nasopharyngeal carcinoma in China. Int. J. Radiat. Oncol. Biol. Phys..

[39] Balachandran V.P., Gonen M., Smith J.J., DeMatteo R.P. (2015). Nomograms in oncology: More than meets the eye. Lancet Oncol..

