# Supplementary material for: External Validation of a Nomogram to Predict Survival and Benefit of Concurrent Chemoradiation for Stage II Nasopharyngeal Carcinoma
Source: Cancers (Basel). 2021 Aug 25;13(17):4286. doi: 10.3390/cancers13174286 (PMC8428339; doi:10.3390/cancers13174286)
Supplement: Supplementary file 1 [file cancers-13-04286-s001.zip › cancers-1356723 - Supplementary.pdf]

Supplementary Materials

Table S1. Patient selection from NPC 1301 study.

Stage II in NPC 1301 study, total, *n*	589
Exclusion Criteria	
	N (%)
Age >70	44 (7.5%)
Incomplete information	4 (0.7%)
Adjuvant chemotherapy	16 (2.7%)
Neoadjuvant chemotherapy	25 (4.2%)
Non-WHO type II or III	18 (3.1%)
No staging MRI	6 (1%)
Non-definitive treatment received	1 (0.17%)

Some patients excluded fulfilled more than one exclusion criterion

Abbreviations: MRI, magnetic resonance imaging; NPC, nasopharyngeal carcinoma; WHO, World Health Organization
